# Silencing LINC00294 Restores Mitochondrial Function and Inhibits Apoptosis of Glioma Cells under Hypoxia via the miR-21-5p/CASKIN1/cAMP Axis

**DOI:** 10.1155/2021/8240015

**Published:** 2021-11-03

**Authors:** Xiaogang Dong, Qin Pi, Anwaier Yuemaierabola, Wenjia Guo, Hailong Tian

**Affiliations:** ^1^Department of Hepatopancreatobiliary Surgery, Cancer Affiliated Hospital of Xinjiang Medical University, Urumqi 830011, China; ^2^Key Laboratory of Oncology of Xinjiang Uyghur Autonomous Region, Urumqi 830011, China; ^3^Department of Cancer Research Institute, Cancer Affiliated Hospital of Xinjiang Medical University, Urumqi 830011, China; ^4^Department of Neurosurgery, Qilu Hospital (Qingdao) of Shandong University, Qingdao, 266000 Shandong, China

## Abstract

Glioma is a type of malignant intracranial tumor. Extensive research has identified the participation of long noncoding RNAs (lncRNAs) in glioma progression. This study investigated the mechanism of LINC00294 in mitochondrial function and glioma cell apoptosis. Glioma miRNA and mRNA microarray datasets were obtained, and differentially expressed lncRNAs in glioma were screened through various databases. The LINC00294 expression in glioma patients and glioma cells was detected. Glioma cells were treated under hypoxic conditions and transfected with LINC00294 silencing. The apoptosis and mitochondrial function of glioma cells were measured. The expressions of and relations among miR-21-5p, CASKIN1, and cAMP in glioma cells were analyzed. Under hypoxic conditions and LINC00294 silencing, the apoptosis and mitochondrial function of glioma cells were detected after inhibiting miR-21-5p or overexpressing CASKIN1. Our results indicated that LINC00294 was downregulated in glioma. LINC00294 silencing inhibited glioma cell apoptosis under hypoxia. LINC00294 silencing reversed the inhibition of hypoxia on mitochondrial function under hypoxia. LINC00294 promoted the CASKIN1 expression by sponging miR-21-5p and activated the cAMP pathway. Inhibition of miR-21-5p or overexpression of CASKIN1 annulled the effects of LINC00294 silencing on mitochondrial function and glioma cell apoptosis under hypoxia. In conclusion, LINC00294 elevated the CASKIN1 expression by sponging miR-21-5p and activating the cAMP signaling pathway, thus inhibiting mitochondrial function and facilitating glioma cell apoptosis.

## 1. Introduction

Glioma is regarded the most common primary intracranial malignancy, which may occur in any part of the central nervous system; however, it elicits predominant manifestation in the brain and glial tissues [1]. Currently, previous exposure to ionizing radiation has been identified as a definitive risk factor for malignant glioma, and additionally, multiple ongoing studies are assessing the genetic risk factors for glioma [2]. The management of glioma presents with enormous clinical challenges, not only due to their characteristic tumor location but also because of their malignant biological characteristics, with a high tendency of proliferation, invasion, angiogenesis, and metabolic abnormality [3]. The currently available medical treatments, including maximal safe resection, external beam radiation, and chemotherapy, can extend the median overall survival of glioma patients up to 12-18 months [4]. Several recent studies have sought to determine the underlying molecular mechanism and biomarkers of glioma for the development of molecular targeted therapies for glioma patients [5]. However, only limited molecular mechanisms have been determined with clinical application. The identification of potent diagnostic and prognostic biomarkers is warranted for effective intervention of glioma.

Recently, the close correlation between oncogene activation and cellular metabolism alteration has been identified [6], where aberrant energy metabolism is also evident in glioma [7]. An existing study elicited mitochondria as vital organelles in diverse cellular functions, such as energy production, reactive oxygen species (ROS), calcium signal transduction, and intermediate biosynthesis [8]. Previously, an association was determined between mitochondrial malfunction and glioma apoptotic signaling [7]. Existing evidence has implicated that unregulated apoptosis may exacerbate uncontrolled tumor occurrence, development, and resistance to chemotherapy and radiotherapy [9]. Moreover, targeting aberrant mitochondrial signaling cascades and apoptosis has been proposed to exhibit paramount efficacy in the management of glioma [10].

Long noncoding RNAs (lncRNAs), a class of noncoding RNA transcripts, expand over more than 200 nucleotides in length that lack protein-coding ability [11]. An existing study identified a vital association between dysregulated lncRNA expression with the pathogenesis of cancers, metabolic disorder, and cardiovascular diseases [12]. Notably, the aberrant lncRNA expression is implicated in glioma initiation and progression, and consequently, lncRNAs can function as competent biomarkers for the diagnosis, prognosis, and target therapy of glioma [13]. Qiu et al. have revealed that LINC00294 facilitates cervical cancer progression by regulation of the cell cycle via the Hedgehog pathway [14]. Notably, the NONCODE database indicated that LINC00294 is upregulated in the normal brain tissues, and GEPIA suggests that LINC00294 is notably downregulated in glioblastoma (GBM) tissues [15]. In light of preceding data, we speculated the definite role and mechanism of LINC00294 in glioma progression. Salmena et al. have proposed a competing endogenous RNA (ceRNA) mechanism in which lncRNA, microRNA (miRNA), and mRNA can interact with each other by the formation of a regulatory network [16]. Existing evidence has elicited the vital functionality of the ceRNA network in the progression and metastasis of human malignancies, including glioma [17]. For instance, lncRNA MATN1-AS1 can evidently facilitate glioma progression by serving as a ceRNA of miR-200b/c/429 [18]. However, whether LINC00294 is implicated in glioma progression through the ceRNA mechanism remains uncertain. Herein, the current study determined LINC00294 interacting partners and elucidated the molecular mechanisms underlying the functions of LINC00294 in glioma, which shall provide a novel theoretical basis for the management of glioma.

## 2. Materials and Methods

### 2.1. Ethics Statement

The study conformed with the ethical guidelines of the Helsinki Declaration and was conducted with approval of the Clinical Ethical Committee of Cancer Affiliated Hospital of Xinjiang Medical University (G-201717) (February 21, 2017). All participants provided written informed consent prior to enrolment.

### 2.2. Bioinformatics Analysis

A combination of miRNA microarray dataset GSE65626 in glioma (containing 3 cancer samples and 3 normal samples) and mRNA microarray dataset GSE50161 (containing 13 cancer samples and 34 normal samples) was identified from the GEO database (https://www.ncbi.nlm.nih.gov/geo/). With the normal samples serving as control, the microarray was differentially analyzed using the limma package in R language and the difference *p* value was adjusted by false discovery rate (FDR). The differentially expressed genes and miRNAs in glioma were screened with the inclusion criteria of ∣logFC | >2 and adj.p.val < 0.05. The ceRNA-related lncRNAs in low-grade glioma (LGG) and GBM collected by TCGA were downloaded from the lncACTdb database (http://www.bio-bigdata.net/LncACTdb/). The downstream miRNAs of LINC00294 were predicted using the starBase database (http://starbase.sysu.edu.cn/). The target genes of miR-21-5p were predicted using the TargetScan database (http://www.targetscan.org/vert_71/) and starBase. The differentially expressed target genes in LGG and GBM collected from TCGA and GTEx were searched through the GEPIA2 database (http://gepia2.cancer-pku.cn/#analysis). The genes related to CASKIN1 were searched on the GeneMANIA database (http://genemania.org/), after which the KEGG pathway enrichment analysis was conducted through the KOBAS 3.0 database (http://kobas.cbi.pku.edu.cn/kobas3/help/). The binding sites of lncRNA-miRNA-mRNA and the coexpression of candidate genes in LGG collected from TCGA were identified using starBase.

### 2.3. Sample Collection

Fifty-seven cases of glioma pathologically confirmed were harvested from patients during the period from January 2014 to January 2016 at the Cancer Affiliated Hospital of Xinjiang Medical University. All patients underwent surgical treatment for the first time, including 37 males and 20 females, aged 49-71 years (an average age of 61 years). According to the 2016 World Health Organization Classification of Tumors of the Central Nervous System [19], 30 cases were classified at stage I-II and 27 cases at stage III-IV and 35 cases with Karnofsky Performance Scale (KPS) score ≥ 70 and 22 cases with KPS score < 70. The exclusion criteria were as follows: patients complicated with other malignant tumors, incomplete clinical data, or severe heart, kidney, and lung dysfunction. Moreover, 24 normal brain tissues were isolated as control from patients who underwent intracranial decompression surgery due to severe craniocerebral injury. No patient received chemoradiotherapy before operation. The 57 patients with glioma were followed up for 5-40 months.

### 2.4. Cell Culture and Transfection

Human microglia CHME-5 and glioma cell lines (SHG44, U251, BT325, and CHG-5 cells) were provided by the Cell Resource Center, Shanghai Institutes for Biological Sciences, Chinese Academy of Sciences (Shanghai, China). The cells were cultured in Dulbecco's modified Eagle's medium (DMEM) containing a combination of 10% fetal bovine serum (FBS), 100 U/mL penicillin, and 100 mg/mL streptomycin at 37°C with 5% CO_2_. The cells were housed in a hypoxia workstation for 24 h with 1% O_2_ [20].

The cells were transfected and assigned into the small hairpin RNA- (shRNA-) negative control (sh-NC) group (cells were transfected with small interfering empty vector), the sh-LINC00294-1~3 group (cells were transfected with lentiviral vectors of sh-LINC00294-1, sh-LINC00294-2, and sh-LINC00294-3), the overexpression- (oe-) NC group (cells were transfected with overexpression empty vector), the oe-CASKIN1 group (cells were transfected with lentiviral vectors of oe-CASKIN1), the mimic NC group (cells were transfected with miR-21-5p mimic NC), the miR-21-5p mimic group (cells were transfected with miR-21-5p mimic), the inhibitor NC group (cells were transfected with miR-21-5p inhibitor NC), and the miR-21-5p inhibitor group (cells were transfected with miR-21-5p inhibitor). The lentiviral vectors were provided by Shanghai Genechem Co., Ltd. (Shanghai, China), and the cells were transfected according to the provided instructions by Genechem. The specified amount of lentivirus (multiplicity of infection = 5) was added into the cell culture plates for cell culture in an incubator at 37°C with 5% CO_2_ for 48 h. The miR-21-5p mimic, mimic NC, miR-21-5p inhibitor, and inhibitor NC were provided by GenePharma (Shanghai, China) and transfected into cells using the Lipofectamine 2000 kit (Invitrogen Inc., Carlsbad, CA, USA). The final concentration of transfection was 100 nM.

In addition, cells in the logarithmic phase were seeded into the 6-well plates and assigned into the Colforsin group (glioma cells were treated with 1.6 *μ*M Colforsin) and the dimethyl sulfoxide (DMSO) group (cells were treated with an equivalent concentration of DMSO) [21]. DMSO and Colforsin were provided by MedChemExpress (HY-15371, Monmouth Junction, NJ, USA).

### 2.5. Reverse Transcription Quantitative Polymerase Chain Reaction (RT-qPCR)

The total RNA content was extracted using the TRIzol reagent (Invitrogen) and reverse transcribed into cDNA using the PrimeScript RT kit (RR037A, Takara, Kyoto, Japan). The reaction system was 10 *μ*L. The reaction solution was utilized for fluorescence quantitative PCR with the SYBR® Premix Ex Taq™ II kit (RR820A, Takara) on the fluorescent quantitative PCR instrument (ABI 7500, ABI, Foster City, CA, USA). Relative expression of the genes was examined based on the 2-^*ΔΔ*Ct^ method, with *β*-actin and U6 serving as internal reference. The experiments were conducted 3 times independently. The primer sequence (Table 1) was synthesized by Sangon Biotech (Shanghai, China).

### 2.6. Fluorescence In Situ Hybridization (FISH)

Specific LINC00294 probes were designed using the Ribo™ lncRNA FISH Probe Mix (Red) kit (RiboBio Co., Ltd, Guangzhou, Guangdong, China). The cover glasses were placed in the 6-well plates, and the glioma cells were seeded on the cover glasses. The cells were cultured under normoxic and hypoxic conditions for 1 day to attain 80% cell confluence. The cover glasses were rinsed with phosphate-buffered saline (PBS), fixed with 1 mL 4% paraformaldehyde, treated with proteinase K (2 *μ*g/mL), glycine, and phthalide reagent, and cultured in 250 *μ*L prehybridization buffer at 42°C for 1 h. Next, after removal of the prehybridization buffer, the cover glasses were cultured in 250 *μ*L hybridization buffer containing probes (300 *μ*g/mL) at 42°C overnight. After a rinse with PBS containing 0.05% (*v*/*v*) Tween-20 (PBST) 3 times, the nuclei were stained with PBST-diluted 4′,6-diamidino-2-phenylindole (DAPI) (1 : 800) for 5 min. After 3 PBST rinses (3 min/time), the cover glasses were sealed with the antifluorescence quencher and observed under a fluorescence microscope (Olympus, Tokyo, Japan).

### 2.7. Flow Cytometry

After 48 h of transfection, the cells were detached with the addition of 0.25% trypsin, collected in flow tube, and centrifuged with removal of the supernatant. The cells were rinsed with PBS 3 times and centrifuged with removal of the supernatant. According to the provided instructions of the Annexin-V-FITC apoptosis detection kit (K201-100, BioVision Inc., Mountain View, CA, USA), the Annexin-V-FITC, propidium iodide (PI), and HEPES buffer were prepared at the ratio of 1 : 2 : 50 into the provided Annexin-V-FITC/PI staining solution. Next, a concentration of 1 × 10^6^ cells was resuspended in per 100 *μ*L stainingsolution and shaken vigorously. After incubation at room temperature for 15 min, 1 mL of the HEPES buffer (PB180325, Procell Life Science & Technology Co., Ltd, Wuhan, Hubei, China) was added to the mixture for vigorous shaking. The fluorescence of FITC and PI was detected with the 525 nm and 620 nm band-pass filters at the excitation wavelength of 488 nm. The cell experiments were conducted 3 times independently.

### 2.8. TUNEL

The cells in the logarithmic growth phase were seeded on the cover glasses in 6-well plates (1 × 10^6^ cells/mL). TUNEL assay was performed with the in situ cell death detection kit (green fluorescence) (11684795910, Roche, Basel, Switzerland). The cell suspension was coated on the cover glasses and fixed with 4% paraformaldehyde for 1 h. Next, the cells were treated with 0.1% Triton X-100 (Beyotime, Shanghai, China) at 4°C for 3 min and cultured with 50 *μ*L of TUNEL in conditions devoid of light at 37°C for 1 h. After 3 PBS rinses, the cells were sealed with the antifluorescence quencher and observed under a fluorescence microscope. Five high-power fields (×400) were randomly selected to count the number of TUNEL-positive cells.

### 2.9. Detection of Mitochondrial DNA (mtDNA) Content

The relative mtDNA content from the total DNA content was determined using RT-qPCR. The relative levels of MT-ND1 gene (F: 5′-CCCTAAAACCCGCCACATCT-3′ and R: 5′-GAGCGATGGTGAGAGCTAAGGT-3′) and nuclear gene human globulin (F: 5′-GTGCACCTGACTCCTGAGGAGA-3′ and R: 5′-CCTTGATACCAACCTGCCCAG-3′) in mtDNA were evaluated.

### 2.10. Detection of ROS

Briefly, a concentration of 3 × 10^5^ cells was seeded into the 6-well plates. After 24 h, the cells were harvested and resuspended using PBS containing 10 *μ*M of CM-H2DCFDA (Invitrogen). The cell-dye mixture was incubated in conditions devoid of light at 37°C for 30 min and then rinsed with PBS prior to the FACS analysis.

### 2.11. Detection of Mitochondrial Membrane Potential (MMP)

MMP was detected by JC-1 under fluorescence microscopy. The JC-1 staining solution was mixed with provided culture medium, added to the samples at the ratio of 5 *μ*L/mL, and placed in the cell culture incubator in conditions devoid of light for 20 min. After 2 rinses, the cells were examined under fluorescence microscopy (Zeiss Inc., AG, Oberkochen, Germany).

### 2.12. Determination of Citrate Synthase Activity

A total of 5 × 10^4^ cells were isolated and rinsed with cold PBS twice. The cell precipitates were dissolved at a concentration of 2 × 10^7^/mL to extract the buffer for incubation on ice for 20 min. After centrifugation at 1600 g for 20 min, the supernatant was transferred to a new tube. Next, 100 *μ*L samples were added to each tube and incubated for 3 h to isolate the enzyme. The suspension was extracted and rinsed twice with 300 *μ*L of 1x washing buffer. Then, 100 *μ*L of 1x citrate synthase (ab119692, Abcam Inc., Cambridge, MA, USA) was added to the samples. The absorbance value at the excitation wavelength of 412 nm was analyzed for 5-30 min at an interval of 20 min on the Synergy 2 Multimode microplate reader (BioTek Instruments Inc., Winooski, VT, USA).

### 2.13. Detection of Adenosine Triphosphate (ATP)

A total of 1 × 10^4^ cells were isolated, incubated in 200 *μ*L cell lysis buffer, and measured using the ATP detection kit (Beyotime). The cells were centrifuged at 1200 g for 5 min, and 100 *μ*L of the supernatant was transferred to the prepared well. The ATP content was measured using the Synergy 2 Multimode microplate reader.

### 2.14. Dual-Luciferase Reporter Gene Assay

The LINC00294 and CASKIN1 3′UTR wild-type (WT) plasmids (CREB1-WT/LINC00294-WT) and the mutant-type (MUT) plasmids (CREB1-MUT/LINC00294-MUT) were methodically constructed. The constructed plasmids were transfected with miR-21-5p mimic and mimic NC into the 293T cells. After 24 h, the cells were lysed and centrifuged at 160000 g for 1 min for isolation of the supernatant. The relative luciferase activity was detected using the Dual-Luciferase® Reporter Assay System (E1910, Promega, Madison, WI, USA). Each cell sample was supplemented with 100 *μ*L firefly luciferase solution to detect the activity of firefly luciferase, and 100 *μ*L renilla luciferase solution was supplemented to detect the activity of renilla luciferase. The relative luciferase activity was estimated as the ratio of firefly luciferase to renilla luciferase. The cell experiments were conducted 3 times independently.

### 2.15. RNA Immunoprecipitation (RIP)

The binding of lncRNA to protein was measured using the RIP kit (Millipore, Billerica, MA, USA). The cells in each group were rinsed with precooled PBS with elimination of the supernatant. The cells were lysed on ice with equal volume of radioimmunoprecipitation assay (RIPA) buffer (P0013B, Beyotime) for 5 min and centrifuged at 220000 g and 4°C for 10 min for isolation of the supernatant. A portion of the cell extract was reserved as input, while the remaining portion was subjected to incubation with the corresponding antibody for coprecipitation. The detailed steps are as follows: 50 *μ*L magnetic beads were rinsed, suspended in 100 *μ*L RIP wash buffer, and incubated with 5 *μ*g rabbit anti-Ago2 (at a dilution ratio of 1 : 100, ab186733, Abcam) for 30 min, with rabbit anti-human IgG (at a dilution ratio of 1 : 100, ab109489, Abcam) as NC. The bead-antibody complex was resuspended in 900 *μ*L RIP wash buffer prior to the overnight incubation with 100 *μ*L of cell extract at 4°C. After 3 rinses, the samples were placed on a magnetic stand for isolation of the magnetic bead-protein complex. The samples and input were detached using protease K treatment to extract the RNA content, which was used for the subsequent detection of LINC00294 and miR-21-5p.

### 2.16. Western Blotting

The tissues or cells detached with trypsin were lysed in RIPA buffer (strong) (BOSTER, Wuhan, Hubei, China) containing a functional proteinase inhibitor and the concentration of protein was detected using the bicinchoninic acid assay kit (BOSTER). The protein was separated on 10% SDS-PAGE and transferred onto polyvinylidene fluoride membranes. A membrane blockade was conducted using 5% bovine serum albumin for 2 h to block any nonspecific binding. Subsequently, the membranes were incubated with the corresponding primary antibodies CASKIN1 (ab2066652, at a dilution ratio of 1 : 1000, Abcam), cAMP (ab235385, at a dilution ratio of 1 : 1000, Abcam), cleaved caspase-3 (ab32042, at a dilution ratio of 1 : 1000, Abcam), cleaved caspase-9 (ab2324, at a dilution ratio of 1 : 1000, Abcam), cleaved caspase-8 (#9496, CST, Beverly, MA, USA), and *β*-actin (ab8226, at a dilution ratio of 1 : 2000, Abcam) at 4°C overnight. Next, the membranes were incubated with the horseradish peroxidase-labeled goat anti-rabbit antibody (ab6721, at a dilution ratio of 1 : 2000, Abcam) for 1 h. The chemiluminescence reagent (Millipore) and X-ray film were used for development and fixation. The gray value of each band was quantified using the ImageJ software, with GAPDH serving as internal control. The cell experiments were conducted 3 times independently.

### 2.17. Statistical Analysis

Data analysis was introduced using the SPSS 21.0 software (IBM Corp., Armonk, NY, USA). The Kolmogorov-Smirnov method was adopted to verify whether the data were in normal distribution. The experimental data were expressed as mean ± standard deviation. The *t-*test was adopted for comparison between two groups. One-way or two-way analysis of variance (ANOVA) was employed for comparisons among multiple groups, followed by Tukey's multiple comparisons test. In all statistical references, a value of *p* < 0.05 was indicative of a statistical difference.

## 3. Results

### 3.1. LINC00294 Was Poorly Expressed in Glioma and Significantly Correlated with Clinical Prognosis

**Figure 1:**
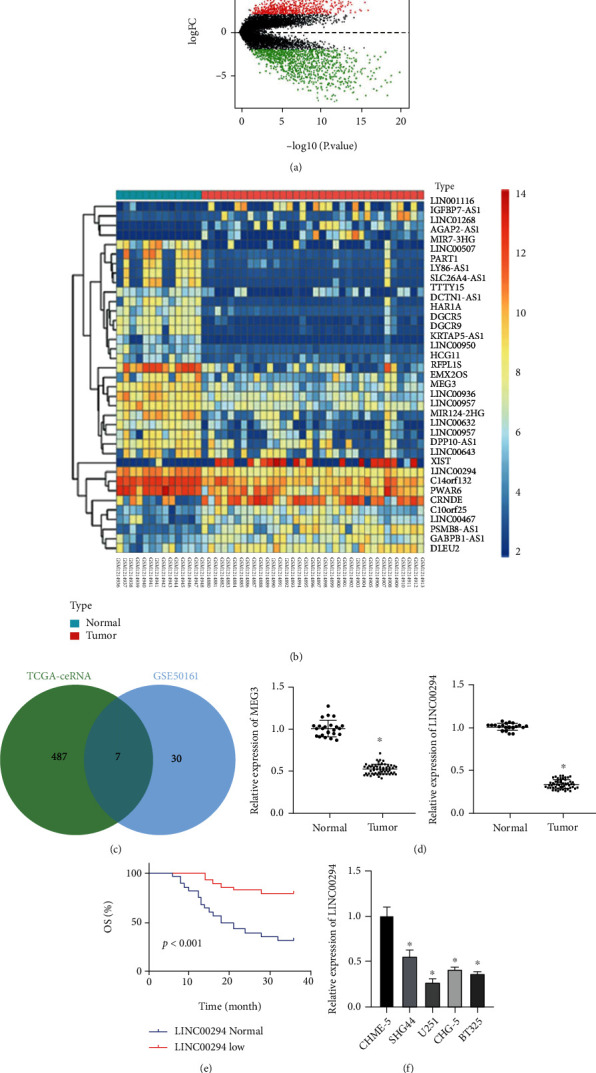
LINC00294 was poorly expressed in glioma and significantly correlated with clinical prognosis. (a) Volcano plot of differentially expressed genes in microarray dataset GSE50161: the abscissa represented −log_10_*p* value and the ordinate represented logFC; the red dots indicated the highly expressed genes in tumor and the green dots indicated the poorly expressed genes in tumor. (b) Heat map of differentially expressed lncRNAs in microarray dataset GSE50161: the abscissa represented the sample number and the ordinate represented the lncRNA name; the left dendrogram showed the clustering of lncRNA expression pattern; each square showed the expression of lncRNA in a sample; the upper right histogram was the color scale. (c) Intersection of the lncRNAs of ceRNA mechanism in TCGA and the differentially expressed lncRNAs in GSE50161, and the middle part represented the intersection of the two sets of data. (d) Expression patterns of MEG3 and LINC00294 in glioma patients were detected using RT-qPCR (normal *N* = 24; tumor *N* = 57). (e) Correlation between LINC00294 expression pattern and clinical survival rate was analyzed. (f) Expression pattern of LINC00294 in microglia and glioma cells was detected using RT-qPCR. The cell experiment was conducted three times independently. Data were expressed as mean ± standard deviation and analyzed using the *t-*test; ^∗^*p* < 0.05*vs*. the normal group or the CHME-5 group.

Microarray dataset GSE50161 in glioma was differentially analyzed, from which 1979 differentially expressed genes were identified (Figure 1(a)). Among these genes, 37 genes revealed a significantly differential expression (Figure 1(b)). The lncRNAs predicted for regulation by ceRNA in TCGA were identified through the lncACTdb database, from which 7 candidate lncRNAs (Supplementary Table 1) were obtained from intersection of predicted results and microarray analysis results (Figure 1(c)). The differential expressions of these 7 lncRNAs in microarray were further searched, and the results indicated the most significant difference between MEG3 and LINC00294 (minimum adj.p.val). In our results, LINC00294 was differentially expressed in the 57 cases of glioma (Figure 1(d)), with a low expression pattern of LINC00294 serving as an indicator of poor prognosis (Figure 1(e)). The LINC00294 expression pattern was reduced in the glioma cells, while U251 cells showed the weakest expression pattern of LINC00294 (Figure 1(f)). Hence, U251 cells were chosen for subsequent experimentation. These results suggested that LINC00294 was poorly expressed in glioma patients and cells and was further associated with clinical prognosis.

### 3.2. LINC00294 Silencing Inhibited Glioma Cell Apoptosis under Hypoxia

**Figure 2:**
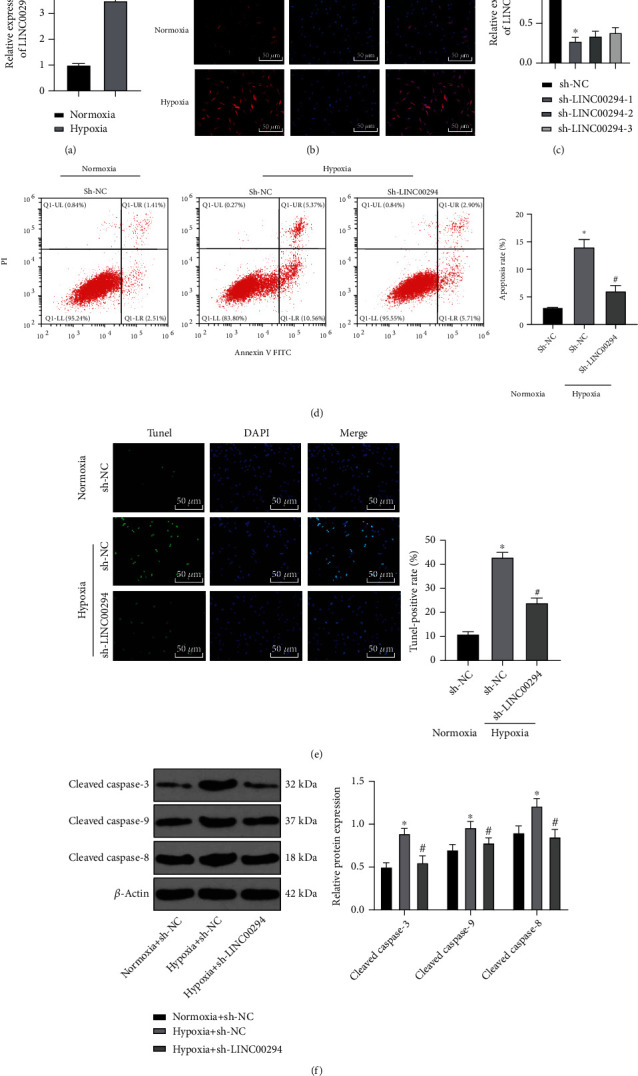
LINC00294 silencing inhibited glioma cell apoptosis under hypoxia. (a) Expression pattern of LINC00294 under normoxia and hypoxia was detected using RT-qPCR. (b) Localization of LINC00294 was detected under normoxia and hypoxia using FISH assay. (c) Transfection efficiency of LINC00294 silencing was confirmed using RT-qPCR. (d, e) Cell apoptosis was measured using flow cytometry and TUNEL. (f) Apoptosis-related proteins (cleaved caspase-3, cleaved caspase-9, and cleaved caspase-8) were detected using Western blotting. The cell experiment was conducted three times independently. Data were expressed as mean ± standard deviation and analyzed using the *t-*test; ^∗^*p* < 0.05*vs*. the normoxia group, the normoxia+sh-NC group, or the sh-NC group; ^#^*p* < 0.05*vs*. the hypoxia+sh-NC group.

Accumulating evidence has elicited that hypoxia induces the apoptosis of glioma cells [22, 23]. The LINC00294 expression pattern under hypoxia and normoxia conditions was detected using RT-qPCR. The results exhibited that the LINC00294 expression pattern was significantly elevated under hypoxia (Figure 2(a)). FISH assay revealed predominant localization of LINC00294 in the cytoplasm, with increased enrichment of LINC00294 under hypoxia (Figure 2(b)). In comparison with the sh-NC-transfected cells, the sh-LINC00294-1- and sh-LINC00294-2-transfected cells showed significantly reduced LINC00294 expression pattern (Figure 2(c)). Thus, sh-LINC00294-1 was chosen for subsequent experimentation because of its superior silencing effect. Next, the LINC00294 expression pattern was silenced in cells under hypoxic condition, with estimation of cell apoptosis by a combination of flow cytometry and TUNEL staining. The results showed that hypoxia induced apoptosis while silencing LINC00294 inhibited glioma cell apoptosis (Figures 2(d) and 2(e)). Western blotting revealed that hypoxia elevated the levels of several apoptosis-related proteins (cleaved caspase-3, cleaved caspase-9, and cleaved caspase-8), and silencing LINC00294 suppressed the levels of these apoptosis-related proteins (Figure 2(f)). Briefly, our results elicited that hypoxia induced glioma cell apoptosis, and silencing LINC00294 inhibited apoptosis.

### 3.3. LINC00294 Silencing Reversed the Inhibition of Mitochondrial Function under Hypoxia

**Figure 3:**
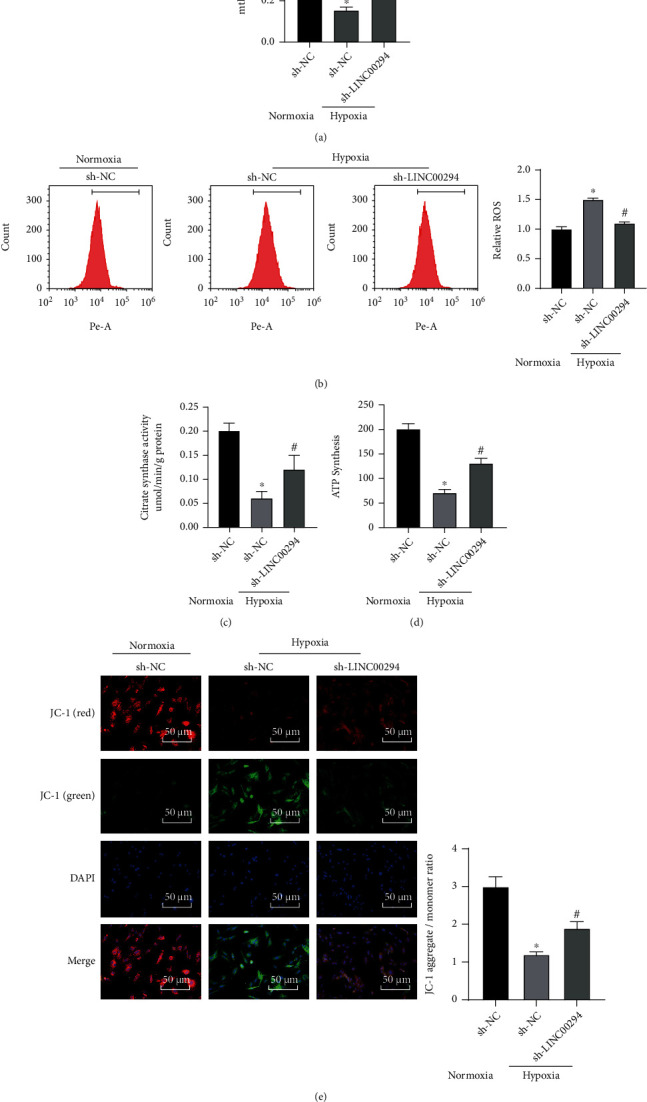
LINC00294 silencing restored mitochondrial function under hypoxia. (a) Level of mtDNA was detected using RT-qPCR. (b) Level of ROS was detected using DCFH-DA fluorescence intensity. (c) Citrate synthase activity was measured by colorimetry. (d) ATP synthesis was analyzed using the ATP synthase assay kit. (e) Alterations of MMP were detected by the MMP detection kit (JC-1). The cell experiment was conducted three times independently. Data were expressed as mean ± standard deviation and analyzed using the *t-*test; ^∗^*p* < 0.05*vs*. the normoxia+sh-NC group; ^#^*p* < 0.05*vs*. the hypoxia+sh-NC group.

ATP deficiency and mitochondrial damage have been proposed as vital causes of cell death in hypoxic glioma [24]. An existing study denoted that ROS accumulation induced by hypoxia has consistently been the primary cause of cell damage; ROS mutilate mitochondrial homeostasis and reduce MMP, resulting in detrimental mitochondrial dysfunction, including decreased ATP synthesis [25]. The mitochondrial function was measured after silencing of LINC00294 under normoxia and hypoxia. Hypoxia significantly reduced the mtDNA level, while LINC00294 silencing elevated the mtDNA level (Figure 3(a)). Our results revealed that hypoxia elevated the ROS level and LINC00294 silencing reduced the ROS level (Figure 3(b)). Hypoxia inhibited the expression pattern of citrate synthase, and LINC00294 silencing increased the expression pattern of citrate synthase (Figure 3(c)). Hypoxia significantly suppressed ATP synthesis and LINC00294 silencing enhanced ATP synthesis (Figure 3(d)). Under hypoxic conditions, MMP mostly presented as green, thus indicative of notably decreased MMP and mitochondrial function, while LINC00294 silencing could restore mitochondrial function (Figure 3(e)). Altogether, our findings elicited that LINC00294 silencing reversed the inhibition of hypoxia on mitochondrial function.

### 3.4. LINC00294 Bound to miR-21-5p

**Figure 4:**
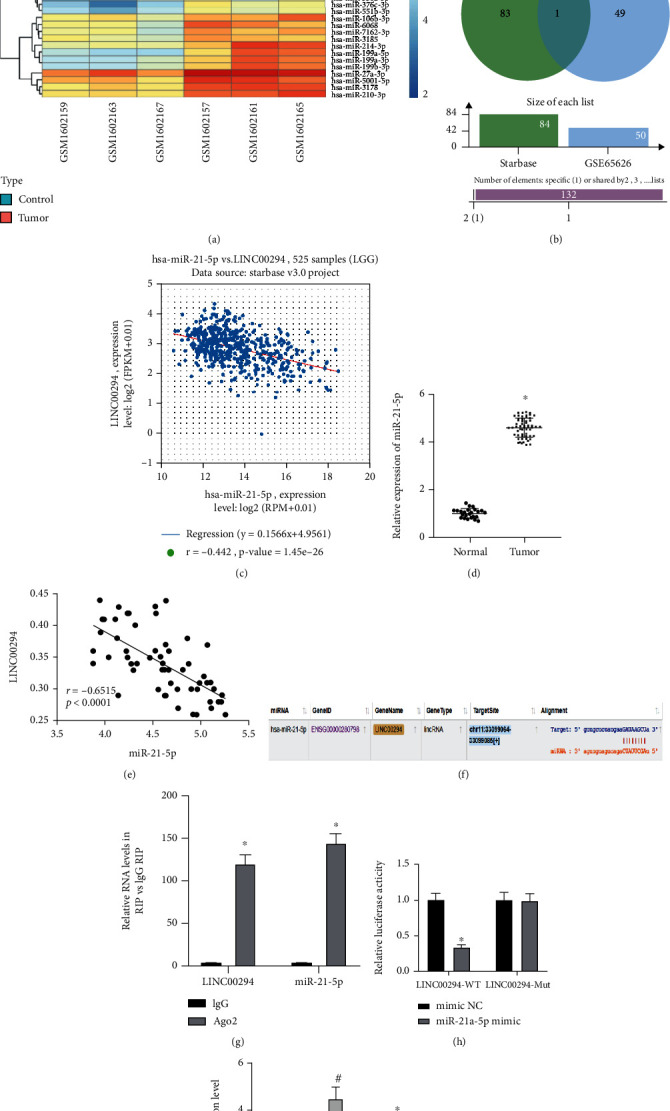
LINC00294 bound to miR-21-5p. (a) Heat map of significantly upregulated miRNAs in GSE65626. (b) Downstream miRNAs of LINC00294 were predicted through starBase. (c) Correlation analysis of miR-21-5p and LINC00294 expression in LGG included in TCGA; the abscissa represented miRNA expression pattern and the ordinate represented lncRNA expression pattern; the upper left represented the correlation coefficient *R* and the correlation *p* value. (d) Expression pattern of miR-21-5p in glioma patients was detected using RT-qPCR. (e) The correlation between LINC00294 and miR-21-5p was analyzed using the Pearson correlation analysis. (f) Binding site of LINC00294 and miR-21-5p was predicted through bioinformatics website. (g) Binding relationship between LINC00294 and miR-21-5p was verified using RIP, with IgG as NC; Ago2 was the core component of RNA inducing complex silencing, which could bind LINC00294 and miR-21-5p. (h) Luciferase activity in each group was detected using dual-luciferase reporter gene assay. (i) Expression patterns of miR-21-5p and LINC00294 were detected using RT-qPCR. The cell experiment was conducted three times independently. Data were expressed as mean ± standard deviation and analyzed using the *t*-test; ^∗^*p* < 0.05*vs*. the normal group, the mimic NC group, the IgG group, or the sh-NC group; ^#^*p* < 0.05*vs*. the oe-NC group.

To determine the downstream mechanism of LINC00294, we differentially analyzed the miRNA microarray dataset GSE65626 in glioma and identified 50 miRNAs with high expressions in glioma (Figure 4(a)). The downstream miRNAs of LINC00294 were predicted through starBase, and the predicted results were intersected with the high expression miRNAs obtained by microarray analysis (Figure 4(b); Supplementary Table 2). From estimation of the intersection results, a candidate miRNA was identified, that is, miR-21-5p. Additionally, our results determined a negative correlation between miR-21-5p and LINC00294 in LGG collected by TCGA (Figure 4(c)). RT-qPCR showed that miR-21-5p was highly expressed in glioma patients (Figure 4(d)). The correlation between LINC00294 and miR-21-5p was analyzed using the Pearson correlation analysis. In the current study, a negative correlation was identified between LINC00294 and miR-21-5p (Figure 4(e)). Bioinformatics website predicted the presence of a binding site between LINC00294 and miR-21-5p (Figure 4(f)). Fundamentally, miRNAs exist in the cytoplasm as miRNA ribonucleoprotein complex, which contains Ago2 as the core component of RNA inducing complex silencing [26]. To verify the binding of LINC00294 to Ago2, RIP analysis was performed on cell extracts using the Ago2 antibody. The results confirmed that LINC00294 and miR-21-5p7 were bound to Ago2 (Figure 4(g)). Dual-luciferase reporter gene assay revealed that the dual-luciferase activity of cells in the miR-21-5p mimic and LINC00294-WT cotransfection group was notably decreased, while no significant difference was evident in the LINC00294-MUT cotransfection group (*p* > 0.05; Figure 4(h)). Our results identified the binding relationship between LINC00294 and miR-21-5p (Figure 4(h)). In comparison with the sh-NC-transfected cells, the sh-LINC00294-transfected cells showed significantly reduced LINC00294 expression pattern and an increased miR-21-5p expression pattern; compared with oe-NC-transfected cells, oe-LINC00294-transfected cells showed an increased LINC00294 expression pattern and reduced miR-21-5p expression pattern (Figure 4(i)). Briefly, our findings determined the binding relationship between LINC00294 and miR-21-5p.

### 3.5. LINC00294 Regulated CASKIN1 Expression by Sponging miR-21-5p and Affected the cAMP Pathway

**Figure 5:**
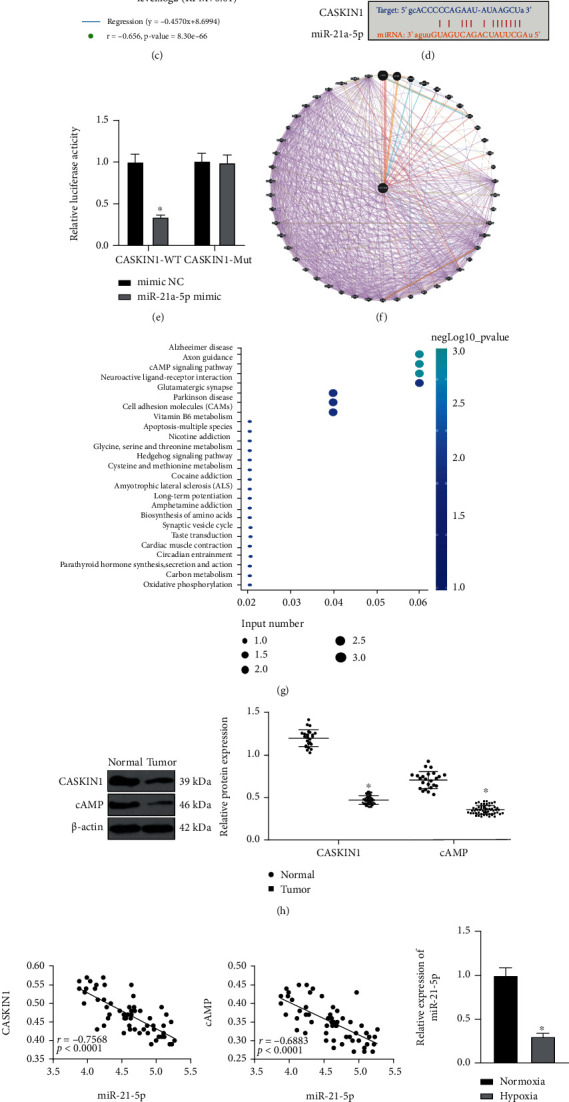
LINC00294 regulated CASKIN1 expression pattern by sponging miR-21-5p and affected the cAMP pathway. (a) Intersection of target genes of miR-21-5p and the significantly downregulated genes in GSE50161, and the middle part represented the intersection of the three sets of data. (b) Differential expression pattern of CASKIN1 in LGG and GBM included in TCGA and GTEx: the abscissa represented the disease type and sample type, and the ordinate represented the expression value; the red box diagram showed the tumor sample, and the gray box diagram showed the normal sample (^∗^*q* < 0.01). (c) Correlation analysis of miR-21-5p and CASKIN1 expression pattern in LGG included in TCGA. (d) Binding site of CASKIN1 and miR-21-5p was predicted through bioinformatics website. (e) Luciferase activity in each group was detected using dual-luciferase reporter gene assay. (f) Prediction of CASKIN1-related genes; the center of the circle was CASKIN1, and the predicted 50 CASKIN1-related genes were surrounded; the connection between genes indicated that there was a correlation ship between genes. (g) Enrichment analysis of CASKIN1-related genes on KEGG pathway; the abscissa represented GeneRatio and the ordinate represented KEGG entry; the size of the circle indicated the number of genes enriched in the entry; the color represented the enriched *p* value, and the histogram on the right was the color scale. (h) Expressions of CASKIN1 and cAMP in glioma patients were detected using Western blotting. (i) The correlations between miR-21-5p and CASKIN1 and cAMP were analyzed using the Pearson correlation analysis. (j) Expression of miR-21a-5p under normoxia and hypoxia was detected using RT-qPCR. (k) Expression patterns of CASKIN1 and cAMP under normoxia and hypoxia were detected using Western blotting. (l) Expression patterns of LINC00294 and CASKIN1 were detected using RT-qPCR. (m) Expression patterns of CASKIN1 and cAMP in each group were detected using Western blotting. The cell experiment was conducted three times independently. Data were expressed as mean ± standard deviation and analyzed using the *t*-test; ^∗^*p* < 0.05*vs*. the normal group, the normoxia group, or the mimic NC group.

The target genes of miR-21-5p were predicted through a combination of starBase and TargetScan, and the predicted results were intersected with the significantly downregulated genes obtained by microarray GSE50161 analysis (Figure 5(a)). Finally, a total of 14 candidate target genes were identified. Among the 14 candidate target genes, CASKIN1 was determined as the most downregulated gene in glioma, with the most significant difference (Supplementary Table 2). Additionally, in our results, CASKIN1 was downregulated in the tumor samples of LGG and GBM collected by TCGA and GTEx (Figure 5(b)). Furthermore, a negative correlation was identified between CASKIN1 and the miR-21-5p expression in LGG samples (Figure 5(c)). Bioinformatics website predicted the presence of a binding site between miR-21-5p and CASKIN1 (Figure 5(d)). Dual-luciferase reporter gene assay revealed that the dual-luciferase activity of cells in the miR-21-5p mimic and CASKIN1-WT cotransfection group was notably decreased, while no significant difference was evident in the CASKIN1-MUT cotransfection group (*p* > 0.05; Figure 5(e)).

Next, a total of 50 genes related to CASKIN1 were identified (Figure 5(f)), and KEGG pathway enrichment analysis was performed on these genes (Figure 5(g)). The results elicited principal enrichment of CASKIN1 related genes in the cAMP pathway. An existing study has implicated the involvement of the cAMP pathway in the regulation of mitochondrial synthesis [27]. In comparison with those of the normal group, the expression patterns of CASKIN1 and cAMP in the tumor group were significantly reduced (Figure 5(h)). The Pearson correlation analysis showed that miR-21-5p was negatively correlated with CASKIN1 and cAMP (Figure 5(i)). Under hypoxia, the miR-21-5p expression pattern was decreased, while the expression patterns of CASKIN1 and cAMP were significantly elevated (Figures 5(j) and 5(k)). To validate the role of miR-21-5p, we detected the expression patterns of LINC00294 and CASKIN1 in cells transfected with miR-21-5p mimic. Relative to the mimic NC-transfected cells, the miR-21-5p mimic-transfected cells showed reduced LINC00294 and CASKIN1 expression patterns (Figure 5(l)), with reduced CASKIN1 and cAMP levels (Figure 5(m)). Briefly, our findings implicated that LINC00294 regulated the CASKIN1 expression by sponging miR-21-5p and influencing the cAMP pathway.

### 3.6. LINC00294/miR-21-5p/CASKIN1 Promoted Glioma Cell Apoptosis under Hypoxia via the cAMP Pathway

**Figure 6:**
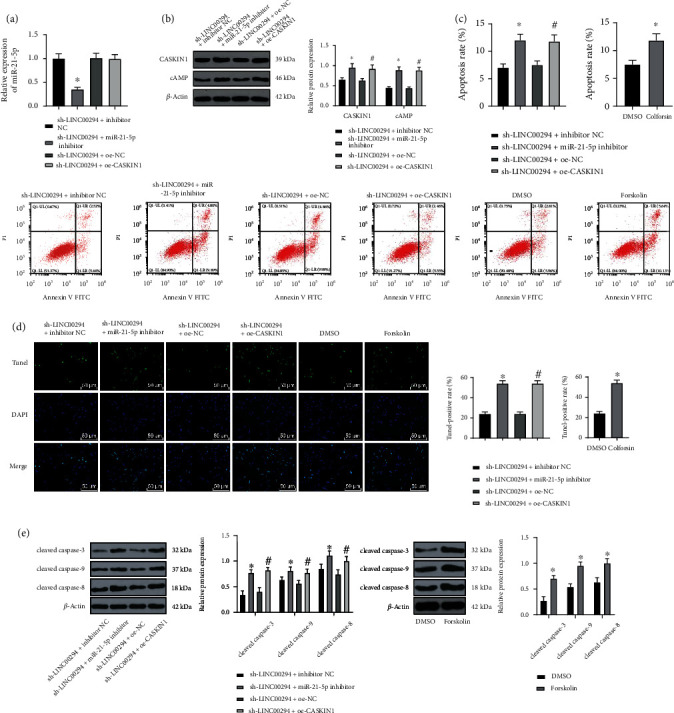
LINC00294/miR-21-5p/CASKIN1 promoted glioma cell apoptosis under hypoxia via the cAMP pathway. (a) Expression pattern of miR-21a-5p under hypoxia was detected using RT-qPCR. (b) Expression patterns of CASKIN1 and cAMP under hypoxia were detected using Western blotting. (c, d) Cell apoptosis was measured using flow cytometry and TUNEL. (e) Apoptosis-related proteins (cleaved caspase-3, cleaved caspase-9, and cleaved caspase-8) were detected using Western blotting. The cell experiment was conducted three times independently. Data were expressed as mean ± standard deviation and analyzed using the *t*-test; ^∗^*p* < 0.05*vs*. the sh-LINC00294+inhibitor NC group; ^#^*p* < 0.05*vs*. the sh-LINC00294+oe-NC group.

To investigate the effect of LINC00294/miR-21-5p/CASKIN1 on glioma cell apoptosis, we transfected experimental cells with the miR-21-5p inhibitor or oe-CASKIN1 under hypoxia and LINC00294 silencing condition. Relative to the sh-LINC00294+inhibitor NC-transfected cells, the sh-LINC00294+miR-21-5p inhibitor-transfected cells showed a lowered miR-21-5p expression pattern, elevated CASKIN1 and cAMP expression patterns, and enhanced apoptosis and apoptosis-related proteins; compared with sh-LINC00294+oe-NC-transfected cells, sh-LINC00294+oe-CASKIN1-transfected cells showed increased CASKIN1 and cAMP expression patterns (Figures 6(a) and 6(b)) with enhanced apoptosis and elevated apoptosis-related proteins (Figures 6(c)–6(e)). The cells were subject to treatment with the cAMP pathway activator (Colforsin). In comparison with the DMSO group, the apoptosis and apoptosis-related proteins in the Colforsin group were significantly elevated (Figures 6(c)–6(e)). These results indicated that LINC00294/miR-21-5p/CASKIN1 promoted glioma cell apoptosis under hypoxia via the cAMP pathway.

### 3.7. LINC00294/miR-21-5p/CASKIN1 Inhibited Mitochondrial Function in Glioma Cells under Hypoxia via the cAMP Pathway

**Figure 7:**
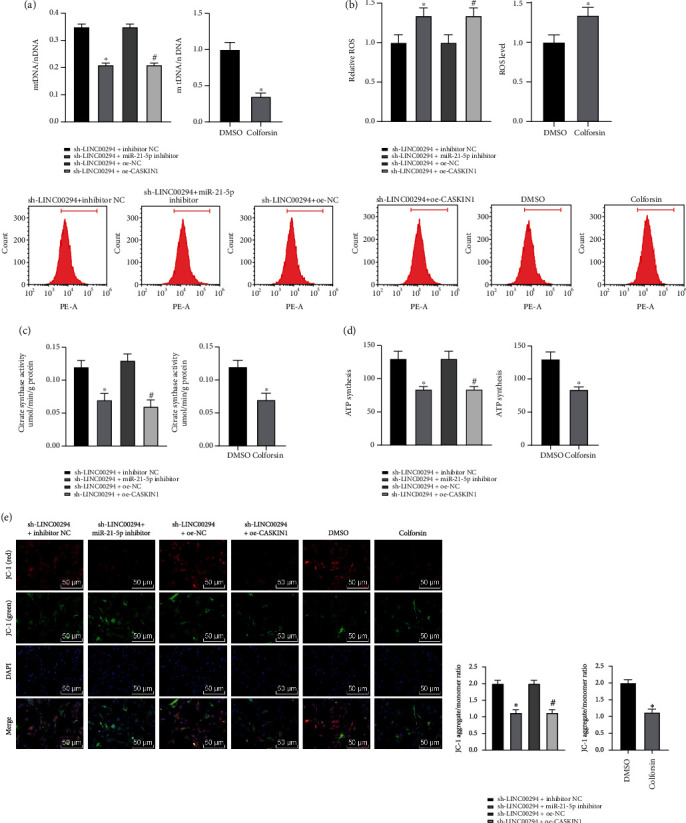
LINC00294/miR-21-5p/CASKIN1 inhibited mitochondrial function in glioma cells under hypoxia via the cAMP pathway. (a) Level of mtDNA under hypoxia was detected using RT-qPCR. (b) Level of ROS under hypoxia was detected using DCFH-DA fluorescence intensity. (c) Citrate synthase activity under hypoxia was measured by colorimetry. (d) ATP synthesis under hypoxia was analyzed using the ATP synthase assay kit. (e) Alterations of MMP under hypoxia were detected by the MMP detection kit (JC-1). The cell experiment was conducted three times independently. Data were expressed as mean ± standard deviation and analyzed using the *t*-test; ^∗^*p* < 0.05*vs*. the sh-LINC00294+inhibitor NC group; ^#^*p* < 0.05*vs*. the sh-LINC00294+oe-NC group.

To study the effect of LINC00294/miR-21-5p/CASKIN1 on the mitochondrial function of glioma cells under hypoxia, we measured the mtDNA level using RT-qPCR. The mtDNA level was notably reduced in cells of the sh-LINC00294+miR-21-5p inhibitor group, the sh-LINC00294+oe-CASKIN1 group, and the Colforsin group, respectively (Figure 7(a)). The ROS level was detected by DCFH-DA fluorescence intensity. Our results revealed that the ROS level of cells was markedly increased in the sh-LINC00294+miR-21-5p inhibitor group, the sh-LINC00294+oe-CASKIN1 group, and the Colforsin group, respectively (Figure 7(b)). The activity of citrate synthetase was determined by colorimetry. The synthesized ATP was analyzed using the ATP synthase detection kit. The alterations in MMP were detected using the MMP detection kit (JC-1). The citrate synthase activity, ATP expression, and JC-1 fluorescence of cells were reduced in the sh-LINC00294+miR-21-5p inhibitor group, the sh-LINC00294+oe-CASKIN1 group, and the Colforsin group, respectively (Figures 7(c)–7(e)). Altogether, our findings revealed that LINC00294/miR-21-5p/CASKIN1 inhibited mitochondrial function in glioma cells under hypoxia via the cAMP pathway.

## 4. Discussion

Glioma is the most frequently diagnosed type of brain tumor and notorious with poor clinical treatment and outcome [28]. Clinically, an abnormal lncRNA expression in glioma samples is associated with malignancy degree and histological differentiation, thus imperative for conclusive diagnosis and prognosis of glioma [29]. The current study demonstrated that a downregulated LINC00294 expression can influence glioma mitochondrial function and cell apoptosis via the ceRNA mechanism.

lncRNAs exhibit functionality via sponging miRNAs, serving as vital ceRNAs to compete with miRNAs for competitive binding to mRNAs for degradation [30]. In our study, the lncRNAs predicted under regulation of the ceRNA mechanism in TCGA were identified using the lncACTdb database. The prediction results were comprehended with the analysis results of glioma microarray GSE50161. Our results revealed that MEG3 and LINC00294 exhibited the most significant difference. Our findings demonstrated that LINC00294 was downregulated in glioma patients, and since downregulation of LINC00294 served as an indicator of poor outcome, we sought to investigate LINC00294. The LINC00294 expression was also reduced in glioma cells, and U251 cells showed the most radically poor expression of LINC00294. Consistently, Zhou et al. have identified a marked reduction in LINC00294 in GBM cells and elicited the potential of overexpressing LINC00294 to repress glioma cell proliferation and trigger apoptosis [15]. Briefly, our findings implicated that LINC00294 was poorly expressed in glioma and was definitively associated with clinical prognosis.

The regulatory balance between cancer cell viability and apoptosis is fundamental for tumorigenesis, and deregulation of apoptosis is a terminal marker of cancer initiation and progression [31]. Hypoxia can trigger the apoptosis of primary, transformed, and cancer cells *in vitro* [24]. Hypoxia is a prevailing characteristic of advanced solid GBM [22]. Our findings revealed that the LINC00294 expression was significantly elevated under hypoxia and a subsequent FISH assay verified the predominant localization of LINC00294 in the cytoplasm. Moreover, the LINC00294 expression in cells was silenced using siRNA under hypoxic conditions, and cell apoptosis was inhibited. The results revealed that LINC00294 silencing could conclusively inhibit glioma cell apoptosis under hypoxia.

Principally, apoptosis can be induced via extrinsic, intrinsic, or mitochondrial pathway [32]. We sought to determine the mitochondrial function of glioma cells under hypoxia. An existing study regarded mtDNA as essential for mitochondrial function; thus, mtDNA deficiency or mutation can result in a variety of diseases [33]. As the product of normal metabolism and exogenous exposure, ROS can induce oxidative modification of cell macromolecules, suppress protein function, and facilitate cell apoptosis [34]. Accumulating evidence has highlighted the vital functionality of citrate synthase in the cell metabolic cycle [35], and a poor citrate synthase expression can facilitate excessive superoxide generation and apoptosis [36]. ATP is actively released in response to tissue injury and cellular stress [37], and MMP reflects the functional status of the mitochondrion [38]. Hypoxia-treated cells elicited reduced mtDNA and MMP levels with inhibited citrate synthase expression and ATP synthesis and an elevated ROS level. Our results indicated that mitochondrial function was radically weakened under hypoxic conditions. In the current study, LINC00294 silencing treatment could restore the mitochondrial function of hypoxia-treated cells. However, due to limited reports on the role of LINC00294 in mitochondrial function, our study presents as a novelty for investigation on LINC00294.

To determine the downstream mechanism of LINC00294, a differential analysis of the miRNA microarray dataset GSE65626 in glioma was conducted. The downstream miRNAs of LINC00294 were predicted through starBase, and the prediction results were intersected with the highly expressed miRNAs obtained by microarray analysis. Finally, a candidate miRNA, miR-21-5p, was identified. Previous literature has highlighted the ability of elevated miR-21-5p expression to facilitate tumor invasion and metastasis of colorectal cancer, breast cancer, and brain cancer [39]. Intriguingly, an elevated miR-21-5p expression has been identified in GBM tissues relative to the reference brain tissues [40]. Subsequently, the target genes of miR-21-5p were predicted through a combination of starBase and TargetScan, and the prediction results were intersected with the significantly downregulated genes obtained by microarray GSE50161 analysis. In the current study, CASKIN1 presented with the most downregulated expression in glioma among the candidate target genes. CASKIN1 principally was identified as a primitive protein that binds to CASK [41]. CASK is a conserved multidomain scaffold protein, which is implicated in brain development, synaptic formation, and cell polarity establishment [42]. Currently, the physiological and pathological roles of CASKIN1 in glioma remain unidentified. We further searched CASKIN1-related genes and analyzed the enrichment of these genes by the KEGG pathway. Our results elicited primary enrichment of the CASKIN1-related genes in the cAMP pathway. An existing study identified that the difference of cAMP pathway activation influences cell susceptibility to malignant transformation and tumorigenesis [43]. Previously, a low level of cAMP could evidently exacerbate gliomagenesis in susceptible Nf1 mice; thus, cAMP-based targeted therapy for glioma has been extensively adopted in mice [44]. In comparison with the normal cells, the tumor cells showed notably reduced CASKIN1 and cAMP expressions. Under hypoxia, the miR-21-5p expression of cells was decreased, while the CASKIN1 and cAMP expressions were significantly elevated. Furthermore, our results revealed reduced expressions of LINC00294, CASKIN1, and cAMP in the miR-21-5p mimic-transfected cells. Briefly, our findings elicited that LINC00294 regulated the CASKIN1 expression by sponging miR-21-5p and influencing the cAMP pathway.

Moreover, we transfected cells with miR-21-5p inhibitor, cAMP signaling pathway activator (Colforsin), or oe-CASKIN1 under hypoxia and LINC00294 silencing condition. The functional rescue experiments verified that the inhibition of miR-21-5p, activation of cAMP pathway, or overexpression of CASKIN1 exacerbated the cell apoptosis and apoptosis-related protein, enhanced ROS level, and decreased citrate synthase activity, ATP synthesis, and JC-1 expression. Previously, the second messenger cAMP has elicited proapoptotic functionality in numerous cell types [45]. Mitochondrial cAMP signal transduction is a vital component of cytoplasmic mitochondrial crosstalk, which regulates mitochondrial homeostasis and mediates mitochondrial dynamics [46]. An investigation by Gulluoglu et al. verified that reduction of miR-21-5p expression can radically exacerbate GBM cell apoptosis [47]. An existing study has implicated the involvement of miR-21-5p in photodynamic therapy-induced glioma cell apoptosis, including the process of inducing ROS content and decreasing MMP expression [48]. Briefly, LINC00294/miR-21-5p/CASKIN1 promoted glioma cell apoptosis and inhibited mitochondrial function via the cAMP pathway.

To conclude, our findings elicited that silencing LINC00294 restored mitochondrial function and inhibited glioma cell apoptosis under hypoxia via the miR-21-5p/CASKIN1/cAMP axis. This fundamental information may hint for utilization of a potential therapeutic target for glioma in the clinical trial. Our further investigations will determine the feasibility and safety of LINC00294 in glioma treatment, in light for the development of LINC00294 from a gene tool into clinical means. Additionally, the mechanism of another differentially expressed lncRNA MEG3 in glioma clinical prognosis, apoptosis, and mitochondrial function warrants further exploration.

## Figures and Tables

**Table 1:** Primer sequence for RT-qPCR.

Primers	Sequences
LINC00294	F: 5′-TGTGTTGTCCTCCAGAATCG-3′
	R: 5′-CCAACCAAGAGCCAACAAAG-3′
MEG3	F: 5′-GGGAGCAGCTATGGATCACC-3′
	R: 5′-ATAGCGCCCCCTATTCATGC-3′
miR-21-5p	F: 5′-TAGCTTATCAGACTGATGTTGA-3′
	R: 5′-TCAACATCAGTCTGATAAGCTA-3′
CASKIN1	F: 5′-GTGGGTCGGAGCCATTCA-3′
	R: 5′-GCCGAGCTGGAGCGTTT-3′
*β*-Actin	F: 5′-AGAGGGAAATCGTGCGTGACA-3′
	R: 5′-CACTGTGTTGGCATAGAGGTC-3′
U6	F: 5′-CTCGCTTCGGCAGCACATATACT-3′
	R: 5′-ACGCTTCACGAATTTGCGTGTC-3′

## Data Availability

All the data generated or analyzed during this study are included in this article.

## Supplementary Materials

Supplementary Materials Supplementary Table 1: the lncRNAs related to ceRNA regulation in TCGA were predicted using the lncACTdb database, and 7 candidate lncRNAs were identified by intersection with differential lncRNAs in the GSE50161 chip. Supplementary Table 2: the target genes of miR-21-5p were predicted using the starBase and TargetScan databases and 14 candidate target genes of miR-21-5p were identified by intersection with the significantly low-expressed genes analyzed by the chip GSE50161. The original figure of binds of Figures 2(f), Figures 5(h), (j), and 5(l), and Figures 6(b) and 6(e).
