# Supplementary material for: Silencing LINC00294 Restores Mitochondrial Function and Inhibits Apoptosis of Glioma Cells under Hypoxia via the miR-21-5p/CASKIN1/cAMP Axis
Source: Oxid Med Cell Longev. 2021 Nov 3;2021:8240015. doi: 10.1155/2021/8240015 (PMC8580631; doi:10.1155/2021/8240015)
Supplement: Supplementary Materials — Supplementary Table 1: the lncRNAs related to ceRNA regulation in TCGA were predicted using the lncACTdb database, and 7 candidate lncRNAs were identified by intersection with differential lncRNAs in the GSE50161 chip. Supplementary Table 2: the target genes of miR-21-5p were predicted using the starBase and TargetScan databases and 14 candidate target genes of miR-21-5p were identified by intersection with the significantly low-expressed genes analyzed by the chip GSE50161. The original figure of binds of Figures 2(f), Figures 5(h), (j), and 5(l), and Figures 6(b) and 6(e). [file 8240015.f1.zip › Supplementary Table 1 (1).docx]

**Supplementary Table 1** Differential expression of candidate lncRNAs and mRNAs in GSE50161

| symbol | logFC | AveExpr | *p*.Value | adj.*p*.Val |
| --- | --- | --- | --- | --- |
| MEG3 (lncRNA) | -3.070831252 | 6.879184716 | 1.34E-11 | 6.69E-10 |
| LINC00294 (lncRNA) | -2.025068556 | 9.207680626 | 2.11E-11 | 9.86E-10 |
| MIR124-2HG (lncRNA) | -4.313095644 | 6.075070366 | 5.68E-09 | 1.10E-07 |
| PSMB8-AS1 (lncRNA) | 3.588936736 | 6.502947382 | 1.14E-08 | 1.99E-07 |
| HCG11 (lncRNA) | -2.002518621 | 4.196543165 | 7.75E-08 | 1.03E-06 |
| DLEU2 (lncRNA) | 2.391999981 | 7.34013327 | 1.45E-05 | 9.55E-05 |
| XIST (lncRNA) | 5.426225619 | 6.606523657 | 0.000688491 | 0.00277676 |
| CASKIN1 | -4.194210843 | -10.81099826 | 3.32E-14 | 5.26E-12 |
| PRKCE | -3.636770867 | -8.910543861 | 1.43E-11 | 7.05E-10 |
| FAM126B | -2.005872251 | -7.703216121 | 8.25E-10 | 2.08E-08 |
| PPP3CA | -2.136907774 | -7.547659665 | 1.40E-09 | 3.27E-08 |
| MKX | -2.895460131 | -7.019050111 | 8.64E-09 | 1.58E-07 |
| ANKRD33B | -3.454784238 | -6.105633902 | 2.03E-07 | 2.38E-06 |
| JPH1 | -3.678750627 | -5.959070313 | 3.36E-07 | 3.67E-06 |
| PAIP2B | -3.612910666 | -5.922663301 | 3.81E-07 | 4.08E-06 |
| NBEA | -2.450080308 | -5.563123807 | 1.31E-06 | 1.20E-05 |
| RASGRP1 | -3.194725447 | -4.519194252 | 4.34E-05 | 0.00024963 |
| EPHA4 | -2.104042348 | -4.230893489 | 0.000110109 | 0.000559773 |
| THRB | -2.815733405 | -4.102276397 | 0.000165657 | 0.000797941 |
| NELL2 | -3.087268228 | -3.947548804 | 0.000269069 | 0.001219752 |
| FOXP2 | -2.821889009 | -3.688928377 | 0.000595091 | 0.002443456 |
