# Supplementary material for: Silencing LINC00294 Restores Mitochondrial Function and Inhibits Apoptosis of Glioma Cells under Hypoxia via the miR-21-5p/CASKIN1/cAMP Axis
Source: Oxid Med Cell Longev. 2021 Nov 3;2021:8240015. doi: 10.1155/2021/8240015 (PMC8580631; doi:10.1155/2021/8240015)
Supplement: Supplementary Materials — Supplementary Table 1: the lncRNAs related to ceRNA regulation in TCGA were predicted using the lncACTdb database, and 7 candidate lncRNAs were identified by intersection with differential lncRNAs in the GSE50161 chip. Supplementary Table 2: the target genes of miR-21-5p were predicted using the starBase and TargetScan databases and 14 candidate target genes of miR-21-5p were identified by intersection with the significantly low-expressed genes analyzed by the chip GSE50161. The original figure of binds of Figures 2(f), Figures 5(h), (j), and 5(l), and Figures 6(b) and 6(e). [file 8240015.f1.zip › Supplementary Table 2 (1).docx]

**Supplementary Table 2** Downstream miRNAs of LINC00294 in Starbase and highly expressed miRNAs in GSE65626

| Starbase | GSE65626 | Starbase\|GSE65626 |
| --- | --- | --- |
| hsa-miR-193a-5p | hsa-miR-10b-5p | hsa-miR-21-5p |
| hsa-miR-2467-3p | hsa-miR-3178 |  |
| hsa-miR-495-3p | hsa-miR-21-3p |  |
| hsa-miR-5688 | hsa-miR-210-3p |  |
| hsa-miR-511-3p | hsa-miR-431-5p |  |
| hsa-miR-541-5p | hsa-miR-424-3p |  |
| hsa-miR-488-3p | hsa-miR-188-5p |  |
| hsa-miR-4677-3p | hsa-miR-3911 |  |
| hsa-miR-4739 | hsa-miR-337-5p |  |
| hsa-miR-4756-5p | hsa-miR-199a-3p |  |
| hsa-miR-1321 | hsa-miR-199b-3p |  |
| hsa-miR-3622b-5p | hsa-miR-6831-5p |  |
| hsa-miR-3611 | hsa-miR-199a-5p |  |
| hsa-miR-196a-5p | hsa-miR-503-5p |  |
| hsa-miR-196b-5p | hsa-miR-378a-5p |  |
| hsa-let-7a-5p | hsa-miR-371b-5p |  |
| hsa-let-7b-5p | hsa-miR-4701-3p |  |
| hsa-let-7c-5p | hsa-miR-24-2-5p |  |
| hsa-let-7d-5p | hsa-miR-27a-3p |  |
| hsa-let-7e-5p | hsa-miR-195-3p |  |
| hsa-let-7f-5p | hsa-miR-214-3p |  |
| hsa-miR-98-5p | hsa-miR-6075 |  |
| hsa-let-7g-5p | hsa-miR-376c-3p |  |
| hsa-let-7i-5p | hsa-miR-551b-3p |  |
| hsa-miR-4458 | hsa-miR-8063 |  |
| hsa-miR-4500 | hsa-miR-5001-5p |  |
| hsa-miR-205-5p | hsa-miR-7162-3p |  |
| hsa-miR-376b-3p | hsa-miR-4634 |  |
| hsa-miR-376a-3p | hsa-miR-1185-2-3p |  |
| hsa-miR-5579-3p | hsa-miR-3195 |  |
| hsa-miR-590-5p | hsa-miR-6068 |  |
| hsa-miR-202-5p | hsa-miR-106b-3p |  |
| hsa-miR-328-3p | hsa-miR-3185 |  |
| hsa-miR-6849-5p | hsa-miR-489-3p |  |
| hsa-miR-3681-5p | hsa-miR-542-5p |  |
| hsa-miR-4766-3p | hsa-miR-940 |  |
| hsa-miR-4525 | hsa-miR-1202 |  |
| hsa-miR-5010-5p | hsa-miR-4436b-5p |  |
| hsa-miR-4428 | hsa-miR-4521 |  |
| hsa-miR-516b-5p | hsa-miR-6877-3p |  |
| hsa-miR-103a-3p | hsa-miR-4669 |  |
| hsa-miR-107 | hsa-miR-572 |  |
| hsa-miR-374c-3p | hsa-miR-4767 |  |
| hsa-miR-1278 | hsa-miR-25-5p |  |
| hsa-miR-543 | hsa-miR-1246 |  |
| hsa-miR-181a-5p | hsa-miR-1290 |  |
| hsa-miR-181b-5p | hsa-miR-550a-3p |  |
| hsa-miR-181c-5p | hsa-miR-378d |  |
| hsa-miR-181d-5p | hsa-miR-6865-3p |  |
| hsa-miR-4262 |  |  |
| hsa-miR-335-5p |  |  |
| hsa-miR-7151-5p |  |  |
| hsa-miR-580-3p |  |  |
| hsa-miR-144-5p |  |  |
| hsa-miR-217 |  |  |
| hsa-miR-6807-3p |  |  |
| hsa-miR-3144-3p |  |  |
| hsa-miR-625-3p |  |  |
| hsa-miR-1306-5p |  |  |
| hsa-miR-138-5p |  |  |
| hsa-miR-4429 |  |  |
| hsa-miR-320a |  |  |
| hsa-miR-320b |  |  |
| hsa-miR-320c |  |  |
| hsa-miR-545-3p |  |  |
| hsa-miR-320d |  |  |
| hsa-miR-708-5p |  |  |
| hsa-miR-28-5p |  |  |
| hsa-miR-3139 |  |  |
| hsa-miR-122-5p |  |  |
| hsa-miR-380-3p |  |  |
| hsa-miR-382-3p |  |  |
| hsa-miR-140-3p |  |  |
| hsa-miR-143-3p |  |  |
| hsa-miR-6088 |  |  |
| hsa-miR-4770 |  |  |
| hsa-miR-1301-3p |  |  |
| hsa-miR-5047 |  |  |
| hsa-miR-499a-5p |  |  |
| hsa-miR-3150a-3p |  |  |
| hsa-miR-6763-5p |  |  |
| hsa-miR-1270 |  |  |
| hsa-miR-620 |  |  |
