# Supplementary figures and images for: Silencing LINC00294 Restores Mitochondrial Function and Inhibits Apoptosis of Glioma Cells under Hypoxia via the miR-21-5p/CASKIN1/cAMP Axis
Source: Oxid Med Cell Longev. 2021 Nov 3;2021:8240015. doi: 10.1155/2021/8240015 (PMC8580631; doi:10.1155/2021/8240015)

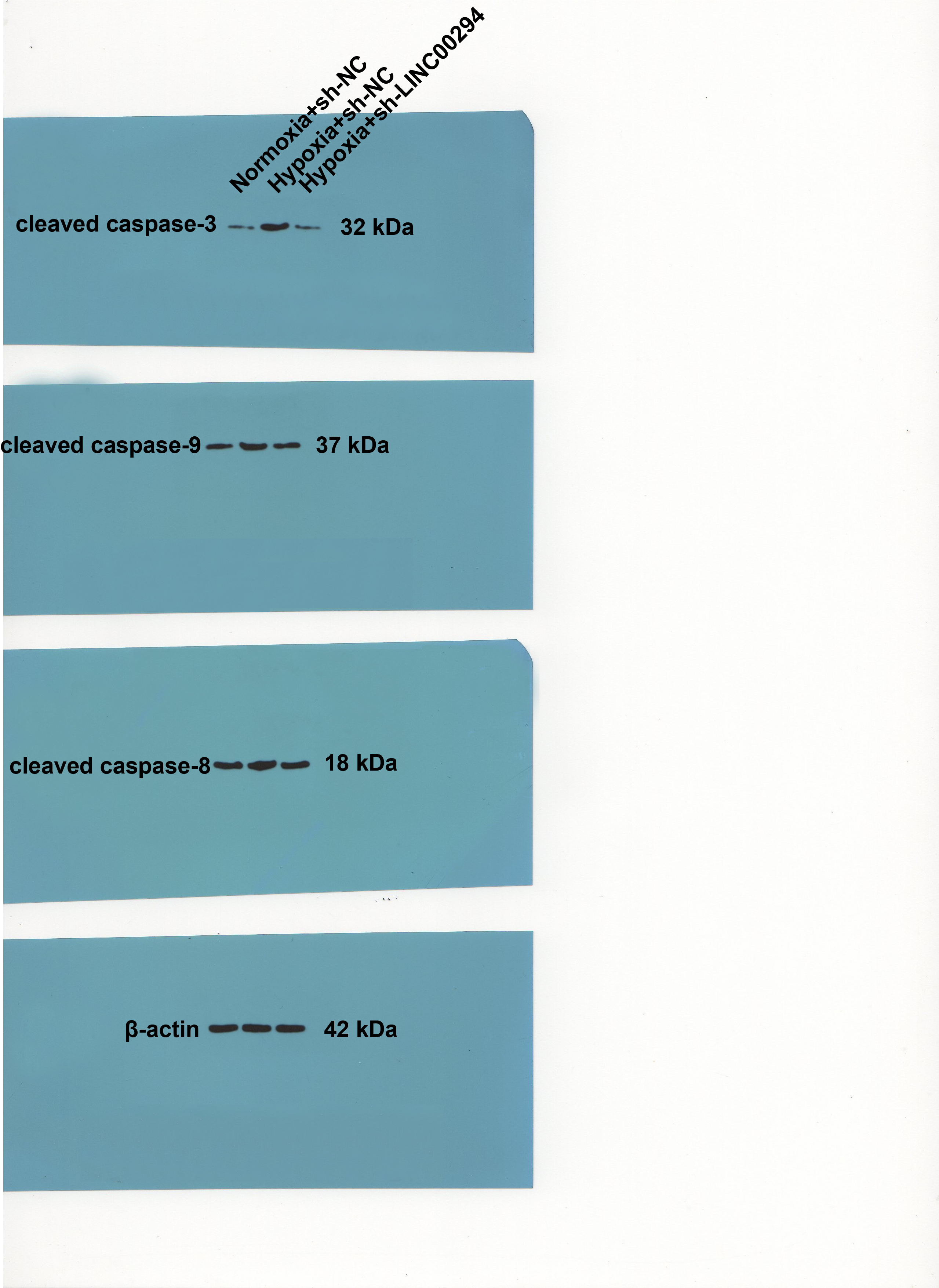

Supplement: Supplementary Materials — Supplementary Table 1: the lncRNAs related to ceRNA regulation in TCGA were predicted using the lncACTdb database, and 7 candidate lncRNAs were identified by intersection with differential lncRNAs in the GSE50161 chip. Supplementary Table 2: the target genes of miR-21-5p were predicted using the starBase and TargetScan databases and 14 candidate target genes of miR-21-5p were identified by intersection with the significantly low-expressed genes analyzed by the chip GSE50161. The original figure of binds of Figures 2(f), Figures 5(h), (j), and 5(l), and Figures 6(b) and 6(e). [file 8240015.f1.zip › original image of figure 2F.jpg]

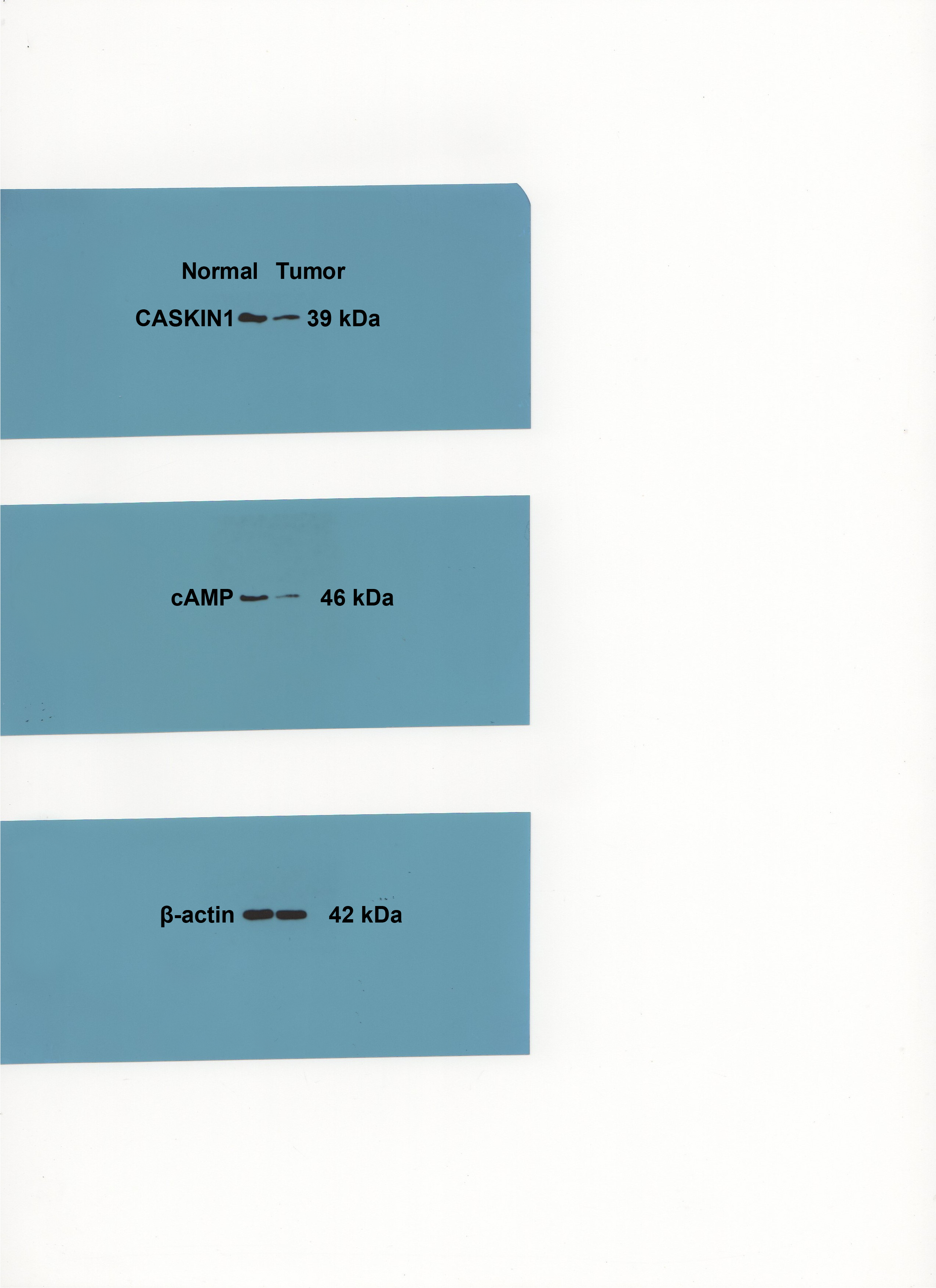

Supplement: Supplementary Materials — Supplementary Table 1: the lncRNAs related to ceRNA regulation in TCGA were predicted using the lncACTdb database, and 7 candidate lncRNAs were identified by intersection with differential lncRNAs in the GSE50161 chip. Supplementary Table 2: the target genes of miR-21-5p were predicted using the starBase and TargetScan databases and 14 candidate target genes of miR-21-5p were identified by intersection with the significantly low-expressed genes analyzed by the chip GSE50161. The original figure of binds of Figures 2(f), Figures 5(h), (j), and 5(l), and Figures 6(b) and 6(e). [file 8240015.f1.zip › original image of figure 5H.jpg]

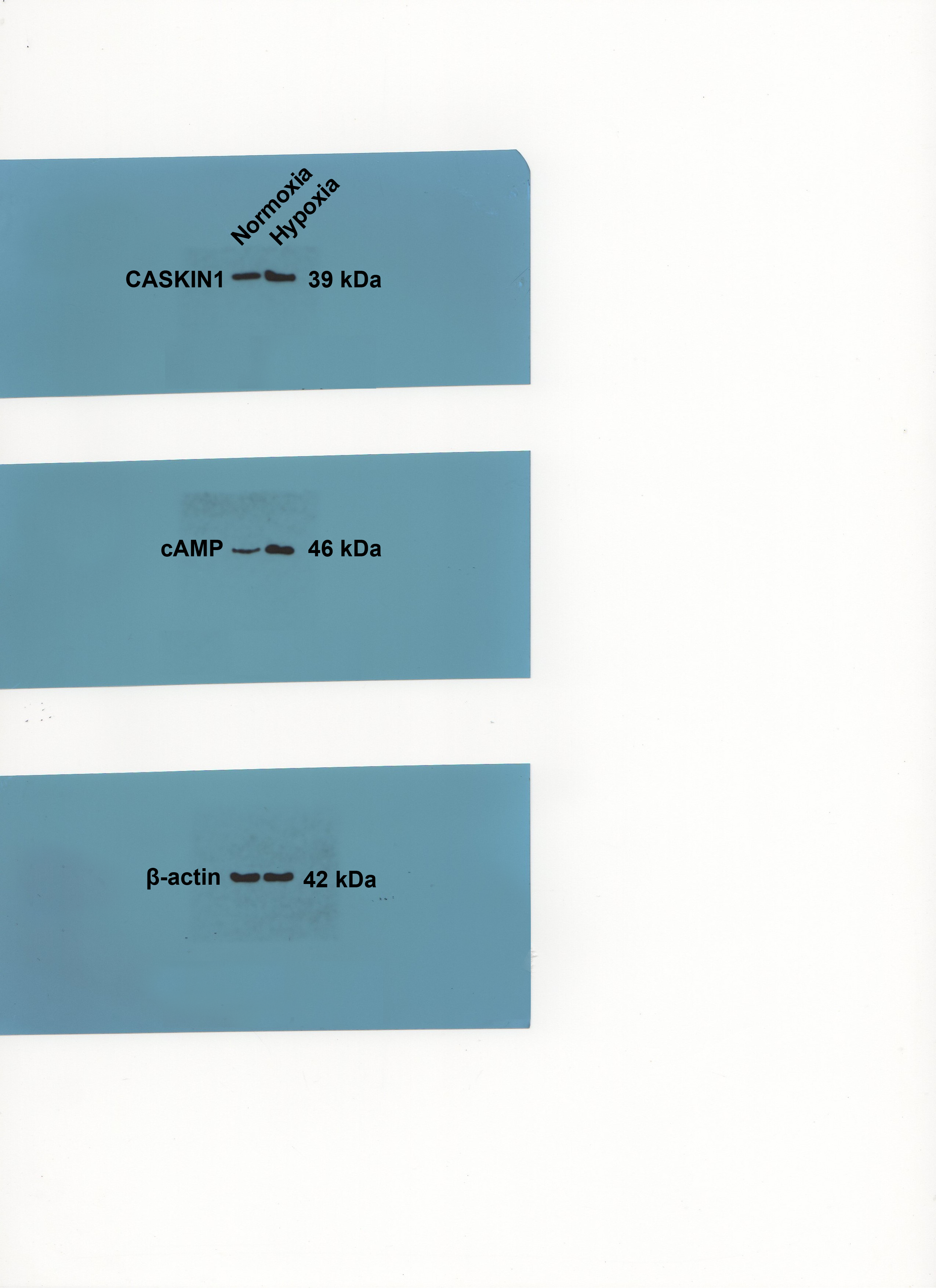

Supplement: Supplementary Materials — Supplementary Table 1: the lncRNAs related to ceRNA regulation in TCGA were predicted using the lncACTdb database, and 7 candidate lncRNAs were identified by intersection with differential lncRNAs in the GSE50161 chip. Supplementary Table 2: the target genes of miR-21-5p were predicted using the starBase and TargetScan databases and 14 candidate target genes of miR-21-5p were identified by intersection with the significantly low-expressed genes analyzed by the chip GSE50161. The original figure of binds of Figures 2(f), Figures 5(h), (j), and 5(l), and Figures 6(b) and 6(e). [file 8240015.f1.zip › original image of figure 5J.jpg]

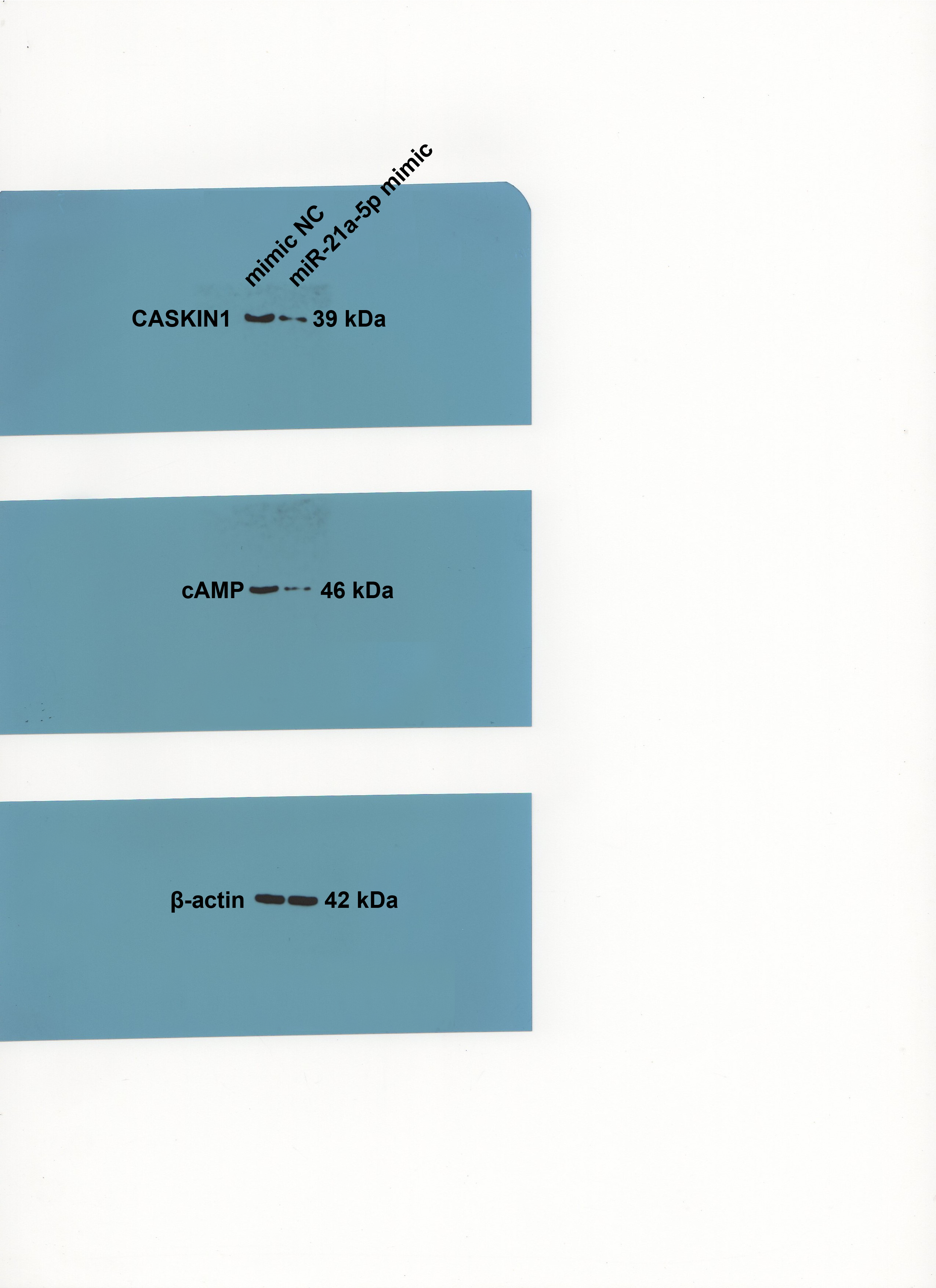

Supplement: Supplementary Materials — Supplementary Table 1: the lncRNAs related to ceRNA regulation in TCGA were predicted using the lncACTdb database, and 7 candidate lncRNAs were identified by intersection with differential lncRNAs in the GSE50161 chip. Supplementary Table 2: the target genes of miR-21-5p were predicted using the starBase and TargetScan databases and 14 candidate target genes of miR-21-5p were identified by intersection with the significantly low-expressed genes analyzed by the chip GSE50161. The original figure of binds of Figures 2(f), Figures 5(h), (j), and 5(l), and Figures 6(b) and 6(e). [file 8240015.f1.zip › original image of figure 5L.jpg]

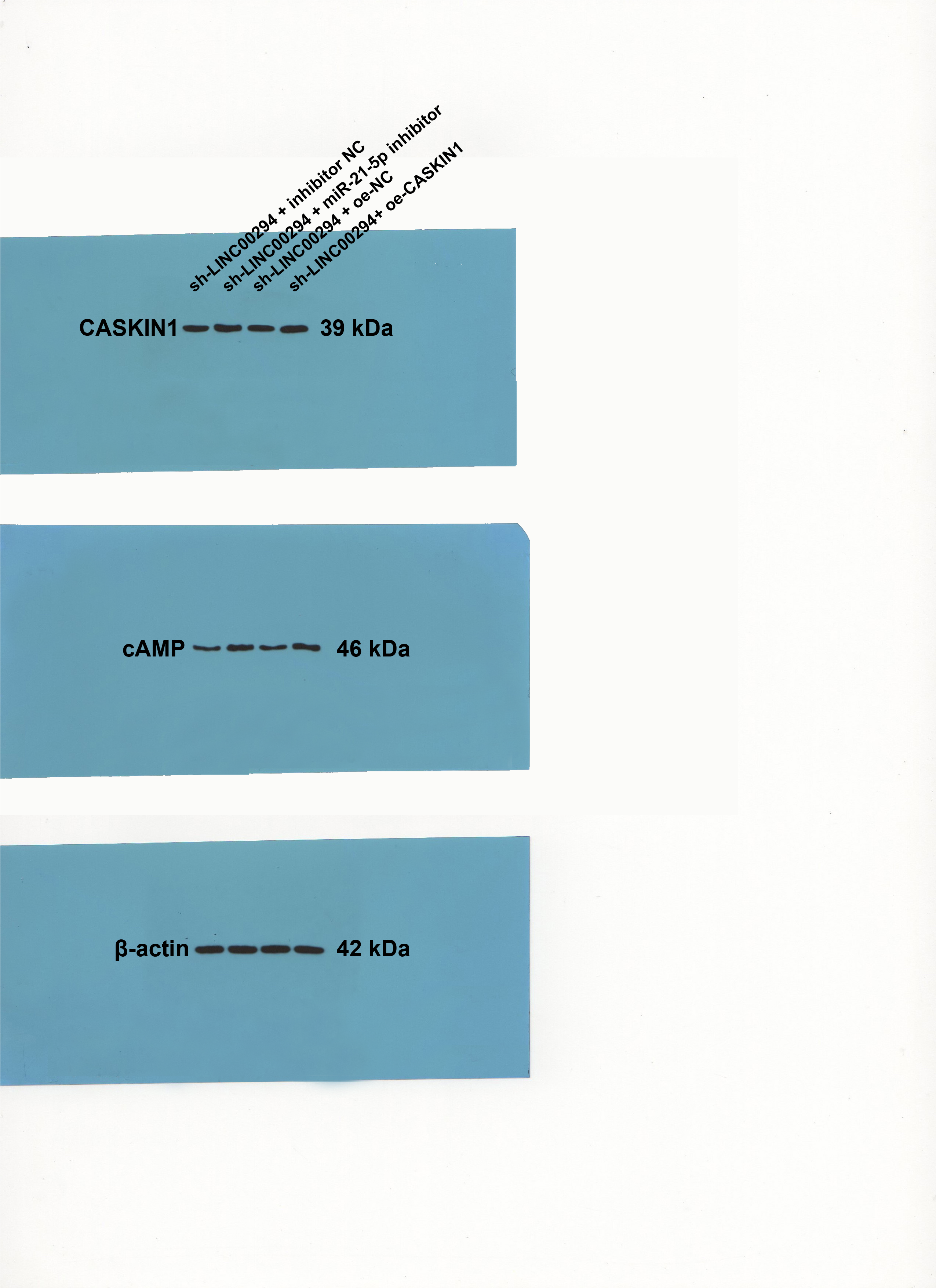

Supplement: Supplementary Materials — Supplementary Table 1: the lncRNAs related to ceRNA regulation in TCGA were predicted using the lncACTdb database, and 7 candidate lncRNAs were identified by intersection with differential lncRNAs in the GSE50161 chip. Supplementary Table 2: the target genes of miR-21-5p were predicted using the starBase and TargetScan databases and 14 candidate target genes of miR-21-5p were identified by intersection with the significantly low-expressed genes analyzed by the chip GSE50161. The original figure of binds of Figures 2(f), Figures 5(h), (j), and 5(l), and Figures 6(b) and 6(e). [file 8240015.f1.zip › original image of figure 6B.jpg]

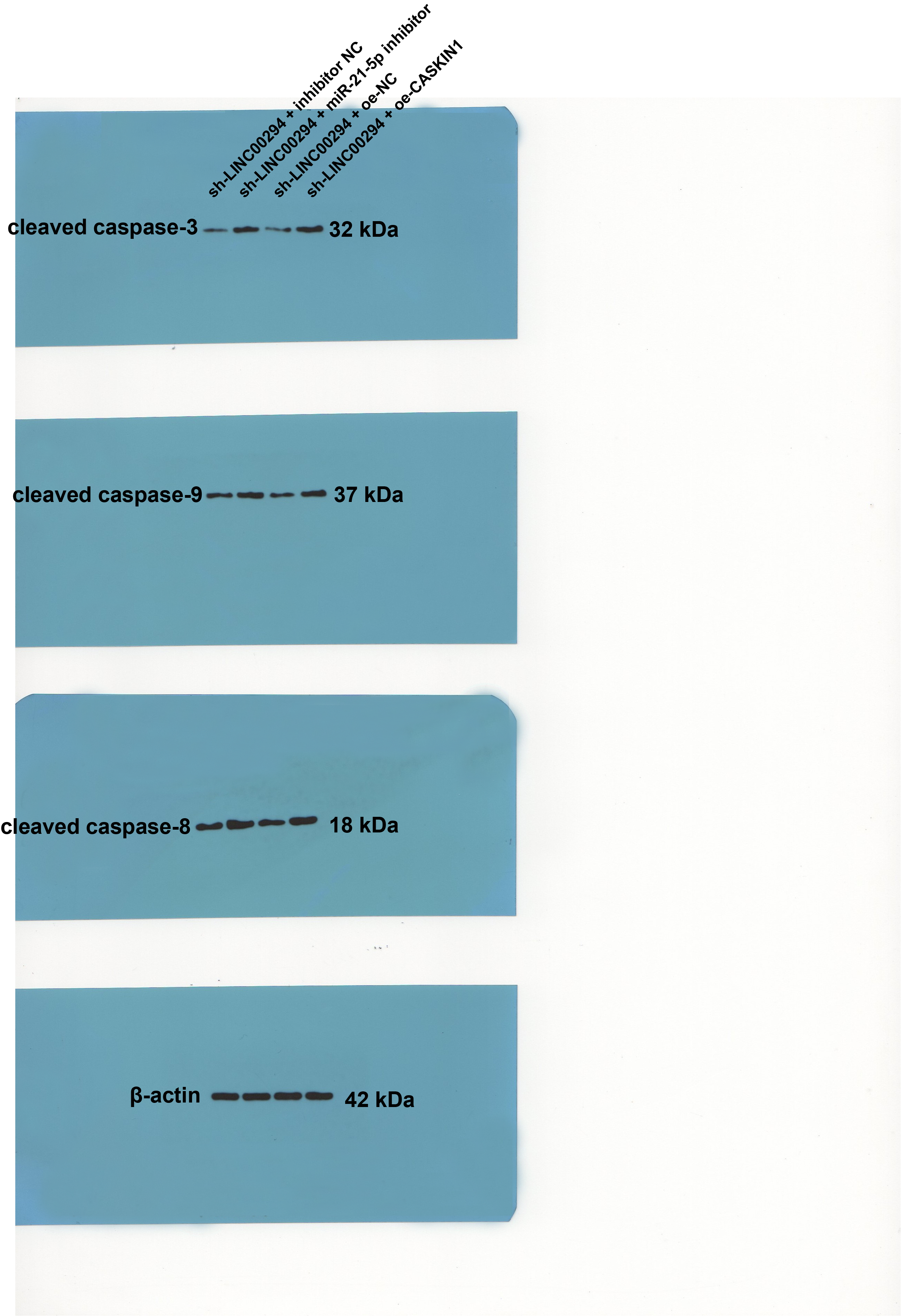

Supplement: Supplementary Materials — Supplementary Table 1: the lncRNAs related to ceRNA regulation in TCGA were predicted using the lncACTdb database, and 7 candidate lncRNAs were identified by intersection with differential lncRNAs in the GSE50161 chip. Supplementary Table 2: the target genes of miR-21-5p were predicted using the starBase and TargetScan databases and 14 candidate target genes of miR-21-5p were identified by intersection with the significantly low-expressed genes analyzed by the chip GSE50161. The original figure of binds of Figures 2(f), Figures 5(h), (j), and 5(l), and Figures 6(b) and 6(e). [file 8240015.f1.zip › original image of figure 6E-1.jpg]

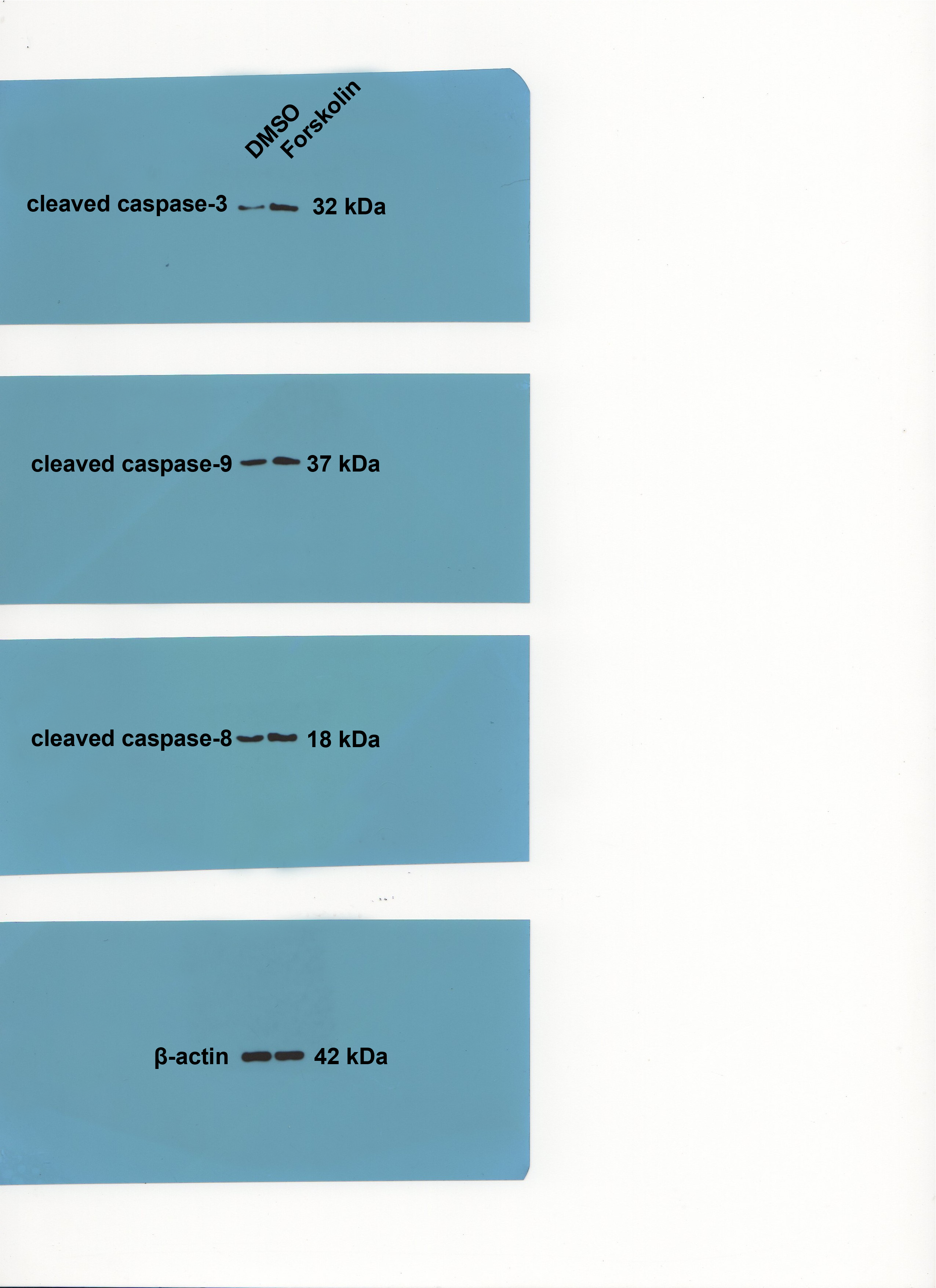

Supplement: Supplementary Materials — Supplementary Table 1: the lncRNAs related to ceRNA regulation in TCGA were predicted using the lncACTdb database, and 7 candidate lncRNAs were identified by intersection with differential lncRNAs in the GSE50161 chip. Supplementary Table 2: the target genes of miR-21-5p were predicted using the starBase and TargetScan databases and 14 candidate target genes of miR-21-5p were identified by intersection with the significantly low-expressed genes analyzed by the chip GSE50161. The original figure of binds of Figures 2(f), Figures 5(h), (j), and 5(l), and Figures 6(b) and 6(e). [file 8240015.f1.zip › original image of figure 6E-2.jpg]
